# Systemic inflammation response index association with gout in hyperuricemic adults: NHANES 2007–2018

**DOI:** 10.3389/fmed.2024.1490655

**Published:** 2025-01-07

**Authors:** Xiaochan Tian, Guixing Zeng, Junping Wei

**Affiliations:** Guang’anmen Hospital, China Academy of Chinese Medical Sciences, Beijing, China

**Keywords:** SIRI, NHANES, the prevalence of gout, hyperuricemic populations, predictive marker, cross-sectional study

## Abstract

**Background:**

Hyperuricemia is the underlying condition of gout. Previous studies have indicated that specific strategies may be effective in preventing the progression of hyperuricemia to gout. However, there is a lack of widely applicable methods for identifying high-risk populations for gout. Gout is linked to inflammation, especially in the hyperuricemic population. Systemic inflammation response index (SIRI) is a novel method for evaluating an individual’s systemic inflammatory activity. However, the association between SIRI and gout in the hyperuricemic population has not been studied.

**Methods:**

The study utilized data from the National Health and Nutrition Examination Survey (NHANES) 2007-2018.SIRI was log2-transformed before analysis. Multivariable logistic regression, subgroup analysis, and smooth curve fitting were employed to comprehensively evaluate the correlation between SIRI and gout prevalence in the hyperuricemic population. Additionally, we compared SIRI with other inflammatory markers.

**Result:**

A total of 6,732 hyperuricemic patients were included, of which 3,764 were men. After adjusting for all covariates, SIRI was found to be significantly positively correlated with gout prevalence in the female group ([OR = 1.385, 95% CI (1.187, 1.615), *p* < 0.001]), and its diagnostic performance was superior to other inflammatory markers. In the male group, the correlation between log2-SIRI and gout prevalence was not significant ([OR = 0.994, 95% CI (0.892, 1.108), *p* = 0.916]). But there were significant positive correlations in the 20–45 age group ([OR = 1.362, 95% CI (1.021, 1.818), *p* = 0.036]). Subgroup analyses revealed that the results were largely consistent when the individuals were divided into different subgroups (FDR adjusted *p* for interaction >0.05 for all).

**Conclusion:**

Our study suggests that the Systemic Inflammation Response Index (SIRI) has potential as a predictive marker for gout risk in hyperuricemic women. However, given the higher gout prevalence in men, the potential of SIRI as a predictive marker for gout risk in this population may be limited. Subgroup analyses, however, indicated that the relationship between SIRI and gout prevalence, as well as its statistical significance, varied across different age groups. Future research could further explore this association by investigating the relationship between SIRI and gout prevalence in different age cohorts.

## Introduction

Gout is an inflammatory arthritis caused by the chronic deposition of monosodium urate crystals. In recent years, with economic development and lifestyle changes, the global prevalence of gout has been on the rise (1, 2). Hyperuricemia is a fundamental condition for gout, with almost all gout patients exhibiting hyperuricemia, but only 20% of those with hyperuricemia develop gout (3). Once gout occurs, it frequently recurs, leading to bone destruction (4) and even joint deformities (5), and increases the risk of various chronic diseases, resulting in significant socioeconomic (6) and personal physical and psychological burdens (7). Current clinical studies indicate that it is possible to prevent the progression from hyperuricemia to gout through urate-lowering therapy or long-term anti-inflammatory treatment (2). However, not all countries have approved urate-lowering therapy for asymptomatic hyperuricemia (8, 9), and anti-inflammatory treatment is not a standard regimen for hyperuricemia. Multiple factors influence the onset of gout, and many of these factors can indicate the risk of gout (10–14). However, objective clinical markers to indicate high-risk individuals for gout among those with hyperuricemia remain lacking.

The systemic inflammation response index (SIRI) is a novel inflammatory marker calculated based on a complete blood cell analysis, reflecting the intensity of the inflammatory response and immune status (15). SIRI has been used to assess the severity and prognosis of various autoimmune diseases, such as rheumatoid arthritis (16), systemic lupus erythematosus (17), and myasthenia gravis (18). Additionally, studies have shown that SIRI has potential applications in evaluating the risk and prognosis of various other systemic diseases (19–24).

The development and progression of gout are closely related to inflammation and immunity (25), which could be a key point for distinguishing high-risk individuals for gout among those with hyperuricemia. Moreover, existing studies have shown that inflammatory markers based on immune cell counts are associated with acute gout flares (26, 27). Therefore, the search for a novel index based on immune cell counts to evaluate the risk of gout in the population with hyperuricemia may hold great promise for preventing gout.

Existing research indicates that SIRI has some value in assessing the disease activity of gout (26) and is both inexpensive and easy to measure. SIRI has the potential to become a predictive marker for gout risk in individuals with hyperuricemia. If a correlation between SIRI and the prevalence of gout is identified in populations with hyperuricemia, it will facilitate the identification of high-risk individuals for gout at a lower economic cost. This would enable targeted early interventions to reduce the incidence of gout, thereby alleviating both the socioeconomic burden and the physiological and psychological burden on patients.

However, due to limited studies, the relationship between SIRI and gout prevalence in hyperuricemic populations remains to be determined. This study aims to analyze the correlation between SIRI and gout prevalence in hyperuricemic individuals using data from the NHANES database to provide a reference for further exploration of SIRI as an inflammatory marker for evaluating gout risk in hyperuricemic populations.

## Materials methods

### Data source and study population

NHANES is a health study conducted by the National Center for Health Statistics (NCHS) at the Centers for Disease Control and Prevention (CDC) aimed at assessing the health and nutritional status of adults and children in the United States. The NHANES has been around for much longer than 10 years. It includes demographic, socioeconomic, dietary, and health-related questions. All data in this study are publicly accessible at http://www.cdc.gov/nchs/nhanes/. All surveys have been approved by NCHS Ethics Review Board (ERB). All individuals included in this study provided informed consent, and the ethical approval from the ethics review board of NCHS can be obtained at https://www.cdc.gov/nchs/nhanes/irba98.htm.

**Figure 1:**
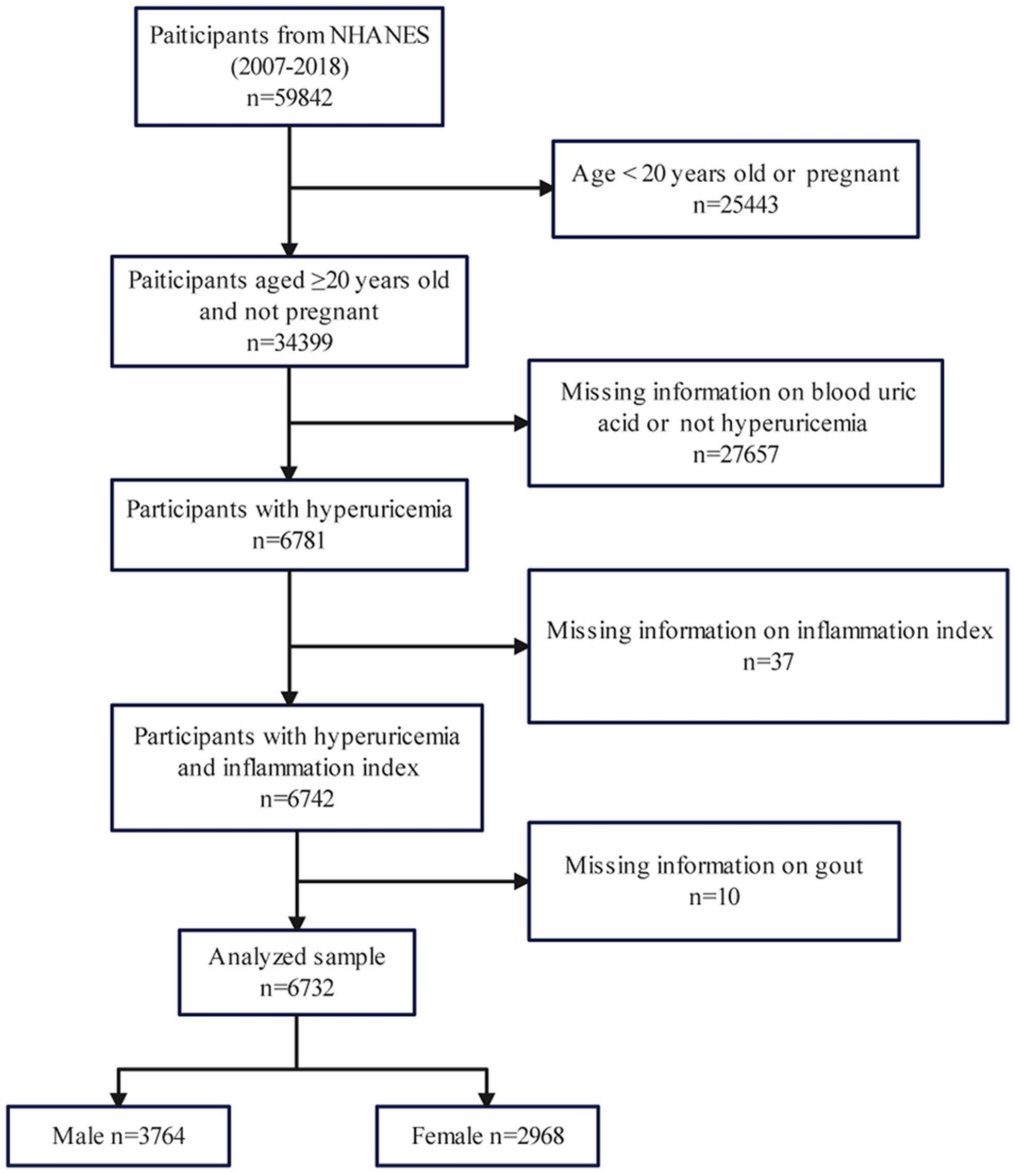
Study flowchart.

Considering the consistency of detection methods of relevant indicators, subjects in this study were selected from the 2007 to 2018 cycle. The inclusion criteria we used for eligible participants were as follows: (1) Aged ≥20 years old and not pregnant; (2) Participants with hyperuricemia; (3) Participants with inflammation index. We also set exclusion criteria: (1) Age < 20 years old or pregnant (*n* = 25,443); (2) Missing information on blood uric acid or not hyperuricemia (*n* = 27,657); (3) Missing information on inflammation index (*n* = 37); (4) Missing information on gout (*n* = 10). Finally, we included 6,732 participants (3,764 male and 2,968 female) out of 59,842 in our research, using the above criteria for further analysis (Figure 1).

### Definition of hyperuricemia and gout

Hyperuricemia was defined as the use of urate-lowering therapy (ULT) or defined based on serum uric acid (SUA). ULT was defined as one of the following medications, either alone or in combination: allopurinol, febuxostat, or probenecid (1). According to SUA levels, hyperuricemia was defined as SUA > 7.0 mg/dL among men OR SUA >6 mg/dL among women (28).

Gout was defined based on a self-reported Medical Conditions questionnaire. We defined gout who answered “yes” to “Has a doctor or other health professional ever told you that you had gout?” (29).

### Inflammatory markers measurement

All inflammatory markers indices were calculated based on the results of complete blood count tests. Plate count (PC), neutrophil count (NC), monocyte count (MC), lymphocyte count (LC), and white blood cell count (WBC) were all expressed in units of 1,000 cells/ml according to the NHANES Laboratory Procedures Manual. SIRI was calculated as NC × MC/LC. In addition, in order to more comprehensively assess the relationship between SIRI and the incidence of gout in hyperuricemic patients, we calculated eight other inflammation-related indices: (1) systemic immune-inflammatory index (SII); (2) total systemic inflammatory index (AISI); (3) platelet-to-lymphocyte ratio (PLR); (4) monocyte-to-lymphocyte ratio (MLR); (5) neutrophil-to-lymphocyte ratio (NLR); (6) product of platelet count and neutrophil count (PPN); (7) derived neutrophil-to-lymphocyte ratio (NLR); (8) neutrophil to monocyte plus lymphocyte ratio (nMLR).

### Normality test

We utilized five statistical tests: the Anderson-Darling normality test, the Cramer-von Mises normality test, the Lilliefors (Kolmogorov–Smirnov) normality test, the Pearson chi-square normality test, and the Shapiro Francia normality test. The normality test is used to test the normality of the inflammatory markers’ distribution. The results showed that the distributions of SIRI and all other inflammatory markers were skewed (Supplementary Table S1) for details. Therefore, the log2-transformation on the SIRI and other inflammatory markers was applied to conduct regression analysis and other analyses.

### Covariates

According to previous studies, the following variables were collected as covariates: sociodemographic characteristics [age, race, education level, and family poverty income ratio (PIR)], medical examination and personal life history (body mass index (BMI), drinking status, diabetes mellitus, hypertension, hyperlipidemia, CKD, physical activity level, serum uric acid, and energy intake). Specific access methods can be found at http://www.cdc.gov/nchs/nhanes/, and detailed definitions and grouping methods can be found in Supplementary Table S2.

### Statistical analysis

The prevalence and characteristics of gout differ between males and females (10). Therefore, analyses were conducted separately for male and female groups. The proportion of missing data for all covariates is at most 30%. We performed imputation using multiple imputation methods.

The baseline characteristics were indicated by number and proportion for categorical variables and mean and standard deviation (SD) for continuous variables. For continuous variables, we calculated *p*-values by doing Kruskal-Wallis rank sum tests. For categorical variables, we computed p-values by doing chi-square tests, and if the number of theoretical frequency l is less than 10, Fisher’s exact probability test was employed.

We used multivariable logistic regression to investigate the association between inflammatory markers and the prevalence of gout in a hyperuricemia population. Model 1 had no covariate adjustments. Model 2 adjusted sociodemographic characteristics for covariates, including age, race, education levels, and PIR. In Model 3, we adjusted for all covariates, including BMI, drinking status, diabetes mellitus, hypertension, hyperlipidemia, CKD, physical activity level, serum uric acid、energy intake, and covariates in Model 2. Moreover, we converted SIRI and other inflammatory markers into quartile variables (Q1–Q4) to assess our findings’ robustness.

Smooth curve fitting was used to address non-linear relationships. Smooth curve fitting was performed using the Generalized Additive Model (GAM) to examine the nonlinear relationship between SIRI and other inflammatory factors with gout prevalence. Gender was a stratifying factor, while age, race/ethnicity, income, education, drinking status, BMI, diabetes, hypertension, hyperlipidemia, physical activity, serum uric acid, and energy intake were adjusted for in the analysis.

Using the ROC curves, the area under the curve was calculated to compare the diagnostic efficacy of the SIRI with other inflammatory markers that correlate significantly with the prevalence of gout.

To explore independent relationships between inflammatory markers SIRI and the prevalence of gout in a hyperuricemia population, we used stratified multivariable logistic regression (Log likelihood ratio test) to adjust covariates. We used the FDR adjustment method to control the false discovery rate in the subgroup analysis.

All analyses were performed by the R software 4.2.1 (https://www.R-project.org) and EmpowerStats2.0 (http://www.empowerstats.com), and statistical significance was set at *p* < 0.05.

## Result

### Baseline characteristics of research participants

Table 1 demonstrates the baseline characteristics of all participants included in the study, including age, ethnicity, educational attainment, PIR, BMI, drinking status, diabetes mellitus, hypertension, hyperlipidemia, CKD, physical activity level, serum uric acid, and energy intake. In both hyperuricemic males and females, participants with gout were prone to having higher SIRI levels, being older, non-Hispanic White, having lower education levels, consuming less alcohol, engaging in less physical activity, having lower serum uric acid levels, having lower energy intake and suffering from diabetes and hypertension (*p* < 0.05). Additionally, male participants were more likely to be wealthier and have a higher prevalence of CKD (p < 0.05).

**Table 1:** Characteristics among hyperuricemic populations with/without gout.

	Total			Male			Female		
Variable	Male	Female	*p*-value	Without gout	With gout	*p*-value	Without gout	With gout	*p*-value
Number	3,764 (55.912%)	2,968 (44.088%)		3,041(80.729%)	723(19.291%)		2,701(91.004%)	267(8.996%)	
Gout	723 (19.208%)	267 (8.996%)	<0.001*						
Sociodemographic variables									
Age, years, *n* (%)			<0.001*			<0.001*			<0.001*
≥20 and <45	1,372 (36.451%)	618 (20.822%)		1,292 (42.486%)	80 (11.065%)		608 (22.510%)	10 (3.745%)	
≥45 and <60	879 (23.353%)	689 (23.214%)		722 (23.742%)	157 (21.715%)		644 (23.843%)	45 (16.854%)	
≥60 and <75	975 (25.903%)	1,041 (35.074%)		678 (22.295%)	297 (41.079%)		917 (33.950%)	124 (46.442%)	
≥75	538 (14.293%)	620 (20.889%)		349 (11.476%)	189 (26.141%)		532 (19.696%)	88 (32.959%)	
Race/ethnicity, *n* (%)			<0.001*			<0.001*			0.035*
Mexican American	426 (11.318%)	310 (10.445%)		382 (12.562%)	44 (6.086%)		290 (10.737%)	20 (7.491%)	
Non-Hispanic White	1,680 (44.633%)	1,292 (43.531%)		1,325 (43.571%)	355 (49.101%)		1,163 (43.058%)	129 (48.315%)	
Non-Hispanic Black	852 (22.635%)	823 (27.729%)		672 (22.098%)	180 (24.896%)		748 (27.693%)	75 (28.090%)	
Other Hispanic	304 (8.077%)	224 (7.547%)		262 (8.616%)	42 (5.809%)		214 (7.923%)	10 (3.745%)	
Other race (including multi-racial)	502 (13.337%)	319 (10.748%)		400 (13.154%)	102 (14.108%)		286 (10.589%)	33 (12.360%)	
Education, *n* (%)			0.325			<0.001*			0.027*
Less than high school	894 (23.751%)	750 (25.270%)		717 (23.578%)	177 (24.481%)		670 (24.806%)	80 (29.963%)	
High school	919 (24.416%)	698 (23.518%)		745 (24.499%)	174 (24.066%)		627 (23.214%)	71 (26.592%)	
More than high school	1951 (51.833%)	1,520 (51.213%)		1,579 (51.924%)	372 (51.452%)		1,404 (51.981%)	116 (43.446%)	
Income, *n* (%)			<0.001*			0.021*			0.069
Poor (<1)	700 (18.597%)	726 (24.461%)		576 (18.941%)	124 (17.151%)		652 (24.139%)	74 (27.715%)	
Near poor (≥1 and ≤3)	1,620 (43.039%)	1,387 (46.732%)		1,331 (43.768%)	289 (39.972%)		1,255 (46.464%)	132 (49.438%)	
Not poor (>3)	1,444 (38.363%)	855 (28.807%)		1,134 (37.290%)	310 (42.877%)		794 (29.397%)	61 (22.846%)	
Medical examination and personal life history									
Drinking status, *n* (%)			<0.001*			<0.001*			0.021*
Never	339 (9.006%)	770 (25.943%)		279 (9.175%)	60 (8.299%)		688 (25.472%)	82 (30.712%)	
Mild	1787 (47.476%)	1,037 (34.939%)		1,394 (45.840%)	393 (54.357%)		933 (34.543%)	104 (38.951%)	
Middle	886 (23.539%)	864 (29.111%)		718 (23.611%)	168 (23.237%)		803 (29.730%)	61 (22.846%)	
heavy	752 (19.979%)	297 (10.007%)		650 (21.375%)	102 (14.108%)		277 (10.255%)	20 (7.491%)	
BMI, *n* (%)			<0.001*			0.269			0.706
Underweight (<18.5)	12 (0.319%)	16 (0.539%)		10 (0.329%)	2 (0.277%)		15 (0.555%)	1 (0.375%)	
Normal (≥18.5 and ≤24.9)	540 (14.346%)	361 (12.163%)		441 (14.502%)	99 (13.693%)		332 (12.292%)	29 (10.861%)	
Overweight (*>*24.9 and <30)	1,284 (34.113%)	696 (23.450%)		1,056 (34.725%)	228 (31.535%)		638 (23.621%)	58 (21.723%)	
Obese (≥30)	1928 (51.222%)	1895 (63.848%)		1,534 (50.444%)	394 (54.495%)		1716 (63.532%)	179 (67.041%)	
Diabetes mellitus, *n* (%)			<0.001*			<0.001*			<0.001*
No	2,827 (75.106%)	1928 (64.960%)		2,403 (79.020%)	424 (58.645%)		1789 (66.235%)	139 (52.060%)	
Yes	937 (24.894%)	1,040 (35.040%)		638 (20.980%)	299 (41.355%)		912 (33.765%)	128 (47.940%)	
Hypertension, *n* (%)			<0.001*			<0.001*			<0.001*
No	1,589 (42.216%)	875 (29.481%)		1,458 (47.945%)	131 (18.119%)		841 (31.137%)	34 (12.734%)	
Yes	2,175 (57.784%)	2093 (70.519%)		1,583 (52.055%)	592 (81.881%)		1860 (68.863%)	233 (87.266%)	
Hyperlipidemia, *n* (%)			<0.001*			0.090			0.212
No	1,043 (27.710%)	1,129 (38.039%)		861 (28.313%)	182 (25.173%)		1,018 (37.690%)	111 (41.573%)	
Yes	2,721 (72.290%)	1839 (61.961%)		2,180 (71.687%)	541 (74.827%)		1,683 (62.310%)	156 (58.427%)	
Chronic kidney disease, *n* (%)			0.472			<0.001*			0.632
No	3,433 (91.206%)	2,692 (90.701%)		2,799 (92.042%)	634 (87.690%)		2,452 (90.781%)	240 (89.888%)	
Yes	331 (8.794%)	276 (9.299%)		242 (7.958%)	89 (12.310%)		249 (9.219%)	27 (10.112%)	
Physical activity (minute/week), mean (SD)			<0.001*			<0.001*			<0.001*
No	2021 (53.693%)	1929 (64.993%)		1,584 (52.088%)	437 (60.443%)		1725 (63.865%)	204 (76.404%)	
Appropriate	931 (24.734%)	782 (26.348%)		747 (24.564%)	184 (25.450%)		729 (26.990%)	53 (19.850%)	
Violent	812 (21.573%)	257 (8.659%)		710 (23.348%)	102 (14.108%)		247 (9.145%)	10 (3.745%)	
Serum uric acid (mg/L), mean ± SD	7.693 ± 1.155	6.934 ± 1.052	<0.001*	7.795 ± 0.835	7.264 ± 1.945	<0.001*	6.926 ± 0.964	7.016 ± 1.704	0.020*
Energy intake (Kcal/day), mean ± SD	2224.240 ± 848.584	1639.883 ± 597.278	<0.001*	2266.873 ± 856.604	2044.923 ± 789.721	<0.001*	1648.754 ± 599.527	1550.146 ± 567.338	0.014*
Immune-inflammation index									
SIRI, mean ± SD	1.441 ± 1.121	1.276 ± 0.932	<0.001*	1.418 ± 1.133	1.538 ± 1.064	<0.001*	1.247 ± 0.901	1.562 ± 1.168	<0.001*
SII, mean ± SD	538.153 ± 568.463	572.221 ± 371.527	<0.001*	535.865 ± 608.031	547.775 ± 357.041	0.332	569.149 ± 369.825	603.292 ± 387.672	0.169
AISI, mean ± SD	339.585 ± 463.686	333.085 ± 280.407	0.710	338.855 ± 496.463	342.657 ± 287.721	0.381	328.265 ± 277.543	381.845 ± 304.175	0.004*
PLR, mean ± SD	119.885 ± 50.805	125.887 ± 52.697	<0.001*	119.488 ± 50.140	121.554 ± 53.515	0.401	126.051 ± 52.430	124.223 ± 55.398	0.376
MLR, mean ± SD	0.315 ± 0.147	0.274 ± 0.130	<0.001*	0.310 ± 0.146	0.335 ± 0.150	<0.001*	0.270 ± 0.126	0.313 ± 0.161	<0.001*
NLR, mean ± SD	2.325 ± 1.410	2.219 ± 1.265	<0.001*	2.286 ± 1.428	2.486 ± 1.323	<0.001*	2.192 ± 1.248	2.488 ± 1.401	<0.001*
PPN, mean ± SD	1060.052 ± 1440.268	1205.429 ± 688.091	<0.001*	1073.536 ± 1578.484	1003.338 ± 562.541	0.015*	1206.072 ± 688.118	1198.923 ± 689.075	0.777
dNLR, mean ± SD	1.555 ± 0.727	1.551 ± 0.716	0.979	1.537 ± 0.730	1.627 ± 0.711	<0.001*	1.540 ± 0.713	1.663 ± 0.745	0.004*
nMLR, mean ± SD	2.640 ± 1.503	2.493 ± 1.345	<0.001*	2.597 ± 1.517	2.821 ± 1.426	<0.001*	2.462 ± 1.326	2.802 ± 1.493	<0.001*

SD, standard deviation; SIRI, systemic inflammatory response index; SII, systemic immune-inflammatory index; AISI, total systemic inflammatory index; PLR, platelet-to-lymphocyte ratio; MLR, monocyte-to-lymphocyte ratio; NLR, neutrophil-to-lymphocyte ratio; PPN, product of platelet count and neutrophil count; dNLR derived neutrophil-to-lymphocyte ratio; nMLR, neutrophil to monocyte plus lymphocyte ratio. *p < 0.05.

### Association of SIRI with the prevalence of gout in a hyperuricemic population and sensitivity analysis

In the hyperuricemic female population (Table 2), under the fully adjusted model (Model 3), log2-SIRI was significantly positively correlated with gout prevalence [OR = 1.385, 95% CI (1.187, 1.615), *p* < 0.001]. This indicates that for each one-unit increase in log2-SIRI, the likelihood of gout increases by 38.5%. Further analysis using log2-SIRI converted into quartiles revealed a significant difference in gout prevalence between the highest and lowest quartiles [OR = 1.867, 95% CI (1.254, 2.778), *p* = 0.002], with a clear trend of increasing gout prevalence associated with higher levels of log2-SIRI (*p* for trend = 0.002). Additionally, significant positive correlations were observed between log2-AISI, log2-MLR, log2-NLR, log2-dNLR, and log2-nMLR with gout prevalence (*p* < 0.05), with a notable trend of increasing gout prevalence associated with higher levels of these inflammatory markers (*p* for trend>0.05).

**Table 2:** Association between SIRI and the prevalence of gout in hyperuricemic female.

Female	Model 1		Model 2		Model 3	
	0R (95%CI)	*p*	0R (95%CI)	*p*	0R (95%CI)	*p*
log2-SIRI	1.448 (1.257, 1.668)	<0.001*	1.423 (1.226, 1.650)	<0.001*	1.385 (1.187, 1.615)	<0.001*
Q1	Reference:1		Reference:1		Reference:1	
Q2	1.265 (0.853, 1.875)	0.243	1.304 (0.870, 1.956)	0.199	1.275 (0.848, 1.917)	0.243
Q3	1.408 (0.956, 2.072)	0.083	1.479 (0.986, 2.217)	0.058	1.352 (0.897, 2.037)	0.150*
Q4	2.066 (1.435, 2.974)	<0.001*	2.040 (1.380, 3.016)	<0.001*	1.867 (1.254, 2.778)	0.002*
*p* for group trend	1.430 (1.202, 1.701)	<0.001*	1.417 (1.178, 1.706)	<0.001*	1.351 (1.118, 1.633)	0.002*
log2-SII	1.103 (0.943, 1.290)	0.220	1.181 (1.009, 1.383)	0.038*	1.140 (0.969, 1.340)	0.114
Q1	Reference:1		Reference:1		Reference:1	
Q2	1.074 (0.741, 1.556)	0.705	1.166 (0.798, 1.702)	0.427	1.118 (0.763, 1.638)	0.568
Q3	1.244 (0.868, 1.784)	0.235	1.436 (0.992, 2.079)	0.055	1.332 (0.917, 1.935)	0.133
Q4	1.263 (0.882, 1.809)	0.203	1.501 (1.033, 2.181)	0.033*	1.379 (0.943, 2.018)	0.097
*p* for group trend	1.158 (0.949, 1.414)	0.148	1.282 (1.042, 1.576)	0.019*	1.220 (0.988, 1.507)	0.065
log2-AISI	1.210 (1.067, 1.373)	0.003*	1.249 (1.096, 1.423)	<0.001*	1.207 (1.055, 1.381)	0.006*
Q1	Reference:1		Reference:1		Reference:1	
Q2	0.962 (0.654, 1.414)	0.844	1.027 (0.692, 1.524)	0.894	0.993 (0.667, 1.477)	0.971
Q3	1.272 (0.883, 1.831)	0.196	1.379 (0.946, 2.008)	0.094	1.253 (0.855, 1.835)	0.247
Q4	1.534 (1.078, 2.184)	0.017*	1.712 (1.178, 2.487)	0.005*	1.543 (1.055, 2.256)	0.025*
*p* for group trend	1.241 (1.063, 1.450)	0.006*	1.300 (1.104, 1.531)	0.002*	1.237 (1.047, 1.462)	0.013*
log2-PLR	0.908 (0.735, 1.124)	0.376	0.925 (0.752, 1.138)	0.460	0.942 (0.762, 1.164)	0.560
Q1	Reference:1		Reference:1		Reference:1	
Q2	1.017 (0.720, 1.436)	0.923	1.114 (0.782, 1.585)	0.551	1.124 (0.787, 1.604)	0.521
Q3	0.921 (0.648, 1.310)	0.648	0.970 (0.677, 1.390)	0.870	0.964 (0.671, 1.385)	0.844
Q4	0.801 (0.557, 1.152)	0.232	0.805 (0.556, 1.166)	0.251	0.833 (0.573, 1.210)	0.338
*p* for group trend	0.831 (0.628, 1.101)	0.197	0.831 (0.625, 1.103)	0.199	0.849 (0.637, 1.132)	0.266
log2-MLR	1.688 (1.377, 2.069)	<0.001*	1.413 (1.138, 1.755)	0.002*	1.433 (1.148, 1.788)	0.001*
Q1	Reference:1		Reference:1		Reference:1	
Q2	1.286 (0.860, 1.923)	0.221	1.200 (0.795, 1.810)	0.386	1.181 (0.781, 1.787)	0.431
Q3	1.318 (0.892, 1.948)	0.166	1.152 (0.770, 1.724)	0.491	1.173 (0.782, 1.760)	0.440
Q4	2.296 (1.597, 3.300)	<0.001*	1.716 (1.162, 2.534)	0.007*	1.698 (1.145, 2.517)	0.008*
*p* for group trend	1.870 (1.441, 2.427)	<0.001*	1.492 (1.127, 1.975)	0.005*	1.485 (1.118, 1.972)	0.006*
log2-NLR	1.408 (1.176, 1.686)	<0.001*	1.376 (1.146, 1.653)	<0.001*	1.344 (1.113, 1.624)	0.002*
Q1	Reference:1		Reference:1		Reference:1	
Q2	1.102 (0.748, 1.624)	0.622	1.155 (0.775, 1.721)	0.480	1.118 (0.748, 1.671)	0.585
Q3	1.373 (0.946, 1.992)	0.096	1.535 (1.040, 2.266)	0.031	1.436 (0.968, 2.128)	0.072
Q4	1.681 (1.175, 2.406)	0.005*	1.653 (1.132, 2.414)	0.009*	1.543 (1.050, 2.269)	0.027*
*p* for group trend	1.435 (1.143, 1.802)	0.002*	1.419 (1.120, 1.797)	0.004*	1.356 (1.064, 1.726)	0.014*
log2-PPN	0.987 (0.841, 1.159)	0.875	1.174 (0.992, 1.390)	0.062	1.094 (0.920, 1.300)	0.310
Q1	Reference:1		Reference:1		Reference:1	
Q2	0.969 (0.679, 1.382)	0.863	1.122 (0.781, 1.610)	0.534	1.078 (0.749, 1.551)	0.687
Q3	1.015 (0.714, 1.442)	0.935	1.228 (0.857, 1.760)	0.262	1.139 (0.791, 1.638)	0.484
Q4	0.936 (0.654, 1.338)	0.715	1.351 (0.930, 1.962)	0.114	1.156 (0.788, 1.695)	0.458
*p* for group trend	0.972 (0.798, 1.184)	0.779	1.190 (0.968, 1.463)	0.098	1.091 (0.883, 1.347)	0.420
log2-dNLR	1.326 (1.078, 1.631)	0.008*	1.367 (1.107, 1.689)	0.004*	1.319 (1.062, 1.639)	0.012*
Q1	Reference:1		Reference:1		Reference:1	
Q2	0.889 (0.602, 1.311)	0.552	0.930 (0.624, 1.385)	0.721	0.924 (0.619, 1.379)	0.698
Q3	1.328 (0.928, 1.902)	0.121	1.472 (1.011, 2.143)	0.044*	1.402 (0.959, 2.048)	0.081
Q4	1.454 (1.022, 2.070)	0.038*	1.531 (1.058, 2.216)	0.024*	1.429 (0.981, 2.081)	0.063
*p* for group trend	1.418 (1.092, 1.841)	0.009*	1.477 (1.126, 1.939)	0.005*	1.395 (1.058, 1.839)	0.018*
log2-nMLR	1.480 (1.225, 1.788)	<0.001*	1.421 (1.171, 1.724)	<0.001*	1.392 (1.140, 1.700)	0.001*
Q1	Reference:1		Reference:1		Reference:1	
Q2	1.055 (0.711, 1.567)	0.790	1.132 (0.754, 1.701)	0.550	1.081 (0.718, 1.629)	0.708
Q3	1.402 (0.964, 2.041)	0.077	1.545 (1.044, 2.287)	0.030*	1.434 (0.965, 2.132)	0.074
Q4	1.832 (1.278, 2.626)	<0.001*	1.767 (1.207, 2.586)	0.003*	1.647 (1.118, 2.426)	0.012*
*p* for group trend	1.573 (1.238, 1.998)	<0.001*	1.516 (1.182, 1.945)	0.001*	1.450 (1.124, 1.870)	0.004*

Model 1: no adjustment. Model 2: adjusted for age, race/ethnicity, income, and education. Model 3: adjusted for age, race/ethnicity, income, education, drinking status, BMI, diabetes mellitus, hypertension, hyperlipidemia, physical activity, serum uric acid, energy intake. OR, odds ratio; CI, confidence interval; SIRI, systemic inflammatory response index; SII, systemic immune-inflammatory index; AISI, total systemic inflammatory index; PLR, platelet-to-lymphocyte ratio; MLR, monocyte-to-lymphocyte ratio; NLR, neutrophil-to-lymphocyte ratio; PPN, product of platelet count and neutrophil count; dNLR derived neutrophil-to-lymphocyte ratio; nMLR, neutrophil to monocyte plus lymphocyte ratio. *p < 0.05.

In the hyperuricemic male population (Table 3), under the fully adjusted model (Model 3), the correlation between log2-SIRI ([OR = 0.994, 95% CI (0.892, 1.108), *p* = 0.916]) and other inflammatory markers’ log-transformed values with gout prevalence was not significant. Among these, log2-NLR, log2-dNLR, and log2-nMLR showed a positive correlation with gout prevalence, while other inflammatory markers were negatively correlated. When converted into quartile variables, the association between log2-SIRI [OR = 0.992, 95% CI (0.861, 1.143), *p* = 0.912] and other inflammatory markers’ quartiles with gout prevalence in the highest quartile compared to the lowest quartile was also not significant. Moreover, the trend of increasing gout prevalence with higher levels of log2-SIRI (p for trend = 0.358) and other inflammatory markers was not significant. In addition, we performed sensitivity analyses by excluding extreme outliers of inflammation markers (Supplementary Table S4) and SUA (Supplementary Table S5). The results remained robust in both the male and female groups.

**Table 3:** Association between SIRI and the prevalence of gout in hyperuricemic female.

Male	Model 1		Model 2		Model 3	
	0R (95%CI)	*p*	0R (95%CI)	*p*	0R (95%CI)	*p*
log2-SIRI	1.046 (0.945, 1.157)	0.388	1.012 (0.913, 1.121)	0.824	0.994 (0.892, 1.108)	0.916
Q1	Reference:1		Reference:1		Reference:1	
Q2	0.845 (0.669, 1.066)	0.155	0.892 (0.698, 1.141)	0.364	0.846 (0.655, 1.092)	0.200
Q3	0.876 (0.695, 1.104)	0.263	0.924 (0.722, 1.182)	0.530	0.881 (0.681, 1.138)	0.332
Q4	1.123 (0.899, 1.403)	0.307	1.000 (0.788, 1.269)	1.000	0.971 (0.757, 1.246)	0.817
*p* for group trend	1.074 (0.945, 1.221)	0.271	1.007 (0.880, 1.152)	0.920	0.992 (0.861, 1.143)	0.912
log2-SII	1.030 (0.949, 1.118)	0.478	0.981 (0.899, 1.070)	0.663	0.958 (0.873, 1.050)	0.358
Q1	Reference:1		Reference:1		Reference:1	
Q2	0.965 (0.765, 1.218)	0.767	0.979 (0.765, 1.254)	0.868	0.878 (0.679, 1.136)	0.322
Q3	1.000 (0.794, 1.260)	1.000	0.951 (0.743, 1.217)	0.687	0.890 (0.688, 1.152)	0.378
Q4	1.143 (0.911, 1.434)	0.248	0.997 (0.779, 1.276)	0.981	0.918 (0.709, 1.188)	0.514
*p* for group trend	1.064 (0.962, 1.178)	0.227	0.996 (0.892, 1.111)	0.941	0.967 (0.862, 1.085)	0.571
log2-AISI	1.177 (1.074, 1.291)	<0.001*	1.012 (0.915, 1.120)	0.809	0.970 (0.872, 1.080)	0.582
Q1	Reference:1		Reference:1		Reference:1	
Q2	0.996 (0.781, 1.270)	0.976	0.955 (0.738, 1.236)	0.726	0.899 (0.688, 1.174)	0.434
Q3	1.446 (1.148, 1.821)	0.002*	1.232 (0.959, 1.584)	0.103	1.123 (0.865, 1.458)	0.385
Q4	1.358 (1.076, 1.713)	0.010*	0.968 (0.748, 1.252)	0.802	0.877 (0.669, 1.148)	0.338
*p* for group trend	1.209 (1.079, 1.354)	0.001*	1.009 (0.889, 1.144)	0.895	0.960 (0.841, 1.096)	0.546
log2-PLR	1.020 (0.882, 1.179)	0.792	0.921 (0.798, 1.063)	0.261	0.977 (0.841, 1.135)	0.759
Q1	Reference:1		Reference:1		Reference:1	
Q2	0.984 (0.780, 1.240)	0.888	0.978 (0.766, 1.250)	0.861	0.970 (0.752, 1.252)	0.817
Q3	0.931 (0.737, 1.177)	0.551	0.939 (0.734, 1.202)	0.618	0.987 (0.764, 1.274)	0.919
Q4	1.192 (0.951, 1.493)	0.127	0.989 (0.780, 1.254)	0.927	1.071 (0.836, 1.373)	0.586
p for group trend	1.144 (0.950, 1.378)	0.156	0.986 (0.812, 1.197)	0.883	1.063 (0.868, 1.301)	0.556
log2-MLR	1.356 (1.181, 1.557)	<0.001*	0.917 (0.789, 1.065)	0.255	0.925 (0.791, 1.082)	0.330
Q1	Reference:1		Reference:1		Reference:1	
Q2	1.106 (0.858, 1.426)	0.435	0.972 (0.743, 1.272)	0.835	0.987 (0.747, 1.303)	0.925
Q3	1.570 (1.239, 1.990)	<0.001*	1.113 (0.863, 1.437)	0.410	1.136 (0.873, 1.478)	0.343
Q4	1.785 (1.409, 2.260)	<0.001*	0.954 (0.733, 1.240)	0.722	0.967 (0.736, 1.269)	0.807
p for group trend	1.634 (1.370, 1.948)	<0.001*	0.980 (0.806, 1.192)	0.841	0.990 (0.808, 1.214)	0.925
log2-NLR	1.294 (1.152, 1.454)	<0.001*	1.072 (0.951, 1.208)	0.256	1.032 (0.909, 1.170)	0.629
Q1	Reference:1		Reference:1		Reference:1	
Q2	1.085 (0.850, 1.385)	0.513	1.084 (0.837, 1.404)	0.542	1.056 (0.808, 1.380)	0.689
Q3	1.363 (1.076, 1.726)	0.010*	1.215 (0.943, 1.566)	0.132	1.129 (0.867, 1.468)	0.368
Q4	1.644 (1.305, 2.070)	<0.001*	1.162 (0.904, 1.495)	0.242	1.083 (0.831, 1.409)	0.556
*p* for group trend	1.419 (1.224, 1.644)	<0.001*	1.109 (0.945, 1.301)	0.205	1.056 (0.892, 1.249)	0.526
log2-PPN	0.876 (0.787, 0.975)	0.015*	1.018 (0.908, 1.141)	0.765	0.972 (0.862, 1.097)	0.651
Q1	Reference:1		Reference:1		Reference:1	
Q2	0.872 (0.697, 1.091)	0.230	0.926 (0.730, 1.173)	0.522	0.838 (0.654, 1.073)	0.161
Q3	0.805 (0.642, 1.011)	0.062	0.933 (0.733, 1.188)	0.574	0.876 (0.681, 1.128)	0.305
Q4	0.782 (0.623, 0.983)	0.035*	1.049 (0.820, 1.341)	0.705	0.956 (0.739, 1.236)	0.730
p for group trend	0.858 (0.749, 0.982)	0.026*	1.025 (0.885, 1.186)	0.745	0.977 (0.839, 1.139)	0.770
log2-dNLR	1.255 (1.096, 1.437)	0.001*	1.100 (0.959, 1.262)	0.171	1.046 (0.907, 1.208)	0.535
Q1	Reference:1		Reference:1		Reference:1	
Q2	0.962 (0.756, 1.224)	0.751	0.989 (0.767, 1.276)	0.932	0.921 (0.707, 1.198)	0.538
Q3	1.158 (0.916, 1.463)	0.219	1.067 (0.831, 1.370)	0.612	0.998 (0.769, 1.295)	0.987
Q4	1.466 (1.169, 1.839)	<0.001*	1.201 (0.939, 1.535)	0.145	1.102 (0.851, 1.427)	0.460
p for group trend	1.380 (1.162, 1.639)	<0.001*	1.163 (0.967, 1.399)	0.108	1.097 (0.903, 1.332)	0.352
log2-nMLR	1.329 (1.175, 1.504)	<0.001*	1.053 (0.924, 1.202)	0.438	1.013 (0.881, 1.165)	0.853
Q1	Reference:1		Reference:1		Reference:1	
Q2	1.188 (0.931, 1.515)	0.167	1.208 (0.932, 1.564)	0.153	1.180 (0.903, 1.542)	0.227
Q3	1.306 (1.027, 1.660)	0.030*	1.145 (0.886, 1.481)	0.301	1.072 (0.821, 1.400)	0.608
Q4	1.773 (1.407, 2.234)	<0.001*	1.231 (0.956, 1.586)	0.108	1.157 (0.887, 1.509)	0.282
p for group trend	1.492 (1.276, 1.744)	<0.001*	1.128 (0.952, 1.337)	0.164	1.078 (0.901, 1.289)	0.412

Model 1: no adjustment. Model 2: adjusted for age, race/ethnicity, income, and education. Model 3: adjusted for age, race/ethnicity, income, education, drinking status, BMI, diabetes mellitus, hypertension, hyperlipidemia, physical activity, serum uric acid, energy intake. OR, odds ratio; CI, confidence interval; SIRI, systemic inflammatory response index; SII, systemic immune-inflammatory index; AISI, total systemic inflammatory index; PLR, platelet-to-lymphocyte ratio; MLR, monocyte-to-lymphocyte ratio; NLR, neutrophil-to-lymphocyte ratio; PPN, product of platelet count and neutrophil count; dNLR derived neutrophil-to-lymphocyte ratio; nMLR, neutrophil to monocyte plus lymphocyte ratio. *p < 0.05.

### Smooth curve fitting

**Figure 2:**
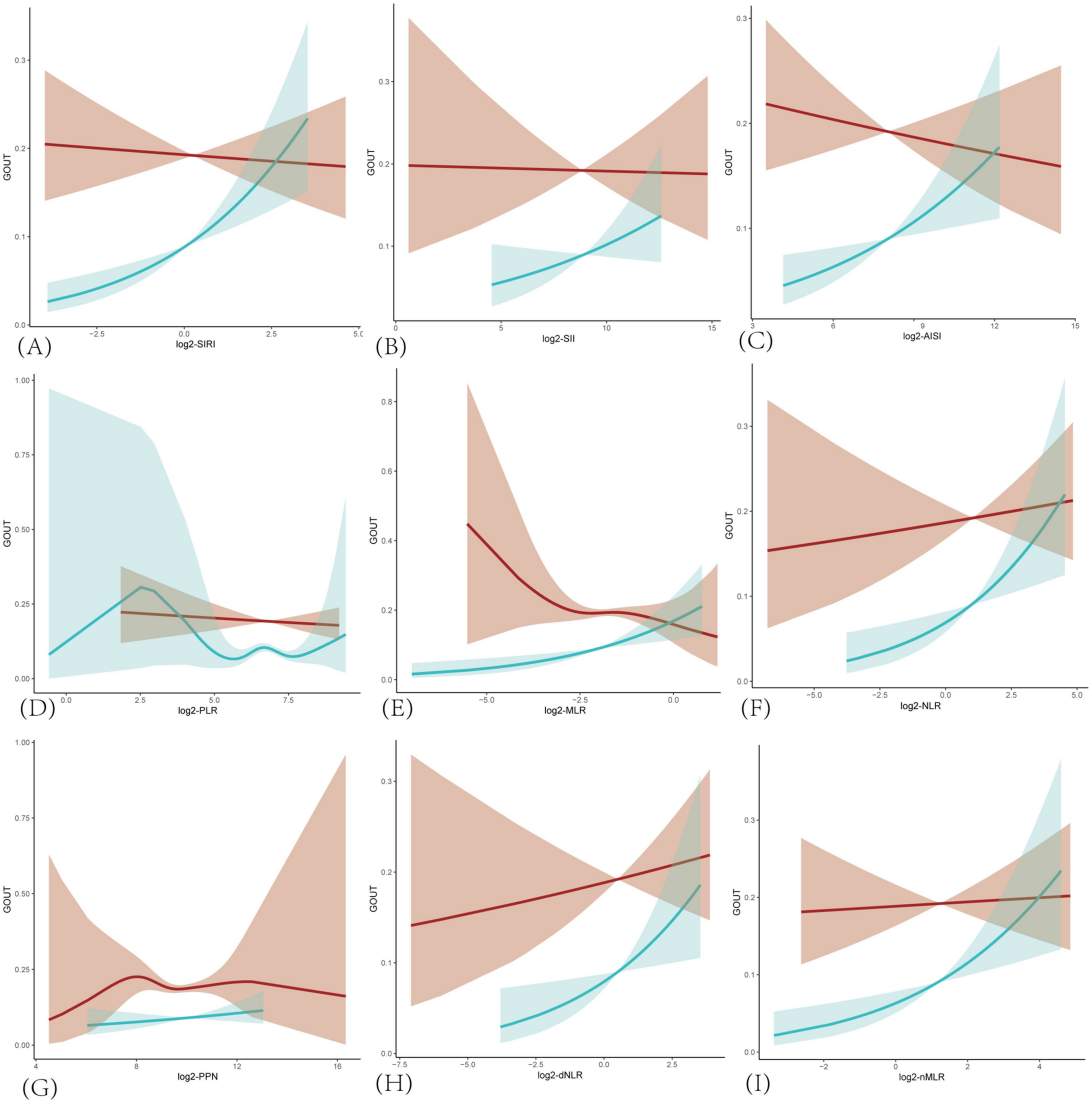
Smoothing curves fitting for the relationship between inflammatory markers and the prevalence of gout in hyperuricemic populations. The red line is for male and blue for female. Adjusted for age, race/ethnicity, income, education, drinking status, BMI, diabetes mellitus, hypertension, hyperlipidemia, physical activity, serum uric acid, energy intake. **(A)** SIRI, systemic inflammatory response index; **(B)** SII, systemic immune-inflammatory index; **(C)** AISI, total systemic inflammatory index; **(D)** PLR, platelet-to-lymphocyte ratio; **(E)** MLR, monocyte-to-lymphocyte ratio; **(F)** NLR, neutrophil-to-lymphocyte ratio; **(G)** PPN, product of platelet count and neutrophil count; **(H)** dNLR derived neutrophil-to-lymphocyte ratio; **(I)** nMLR, neutrophil to monocyte plus lymphocyte ratio.

In addition, smooth curve fitting revealed non-linear relationships between the prevalence of gout and SIRI, as well as other inflammatory markers in the hyperuricemic population (Figure 2). Consistent with the correlation analysis results, the trend of correlation between each inflammatory marker and gout was positive in the female group. In contrast, in the male group, only log2-NLR, log2-dNLR, and log2-nMLR showed a positive correlation, while the other markers were negatively correlated with gout prevalence. Furthermore, log2-PLR in the female group and log2-MLR and log2-PPN in the male group exhibited more complex non-linear relationships with gout prevalence.

### ROC curve analysis and comparison of area under the curve (AUC)

**Figure 3:**
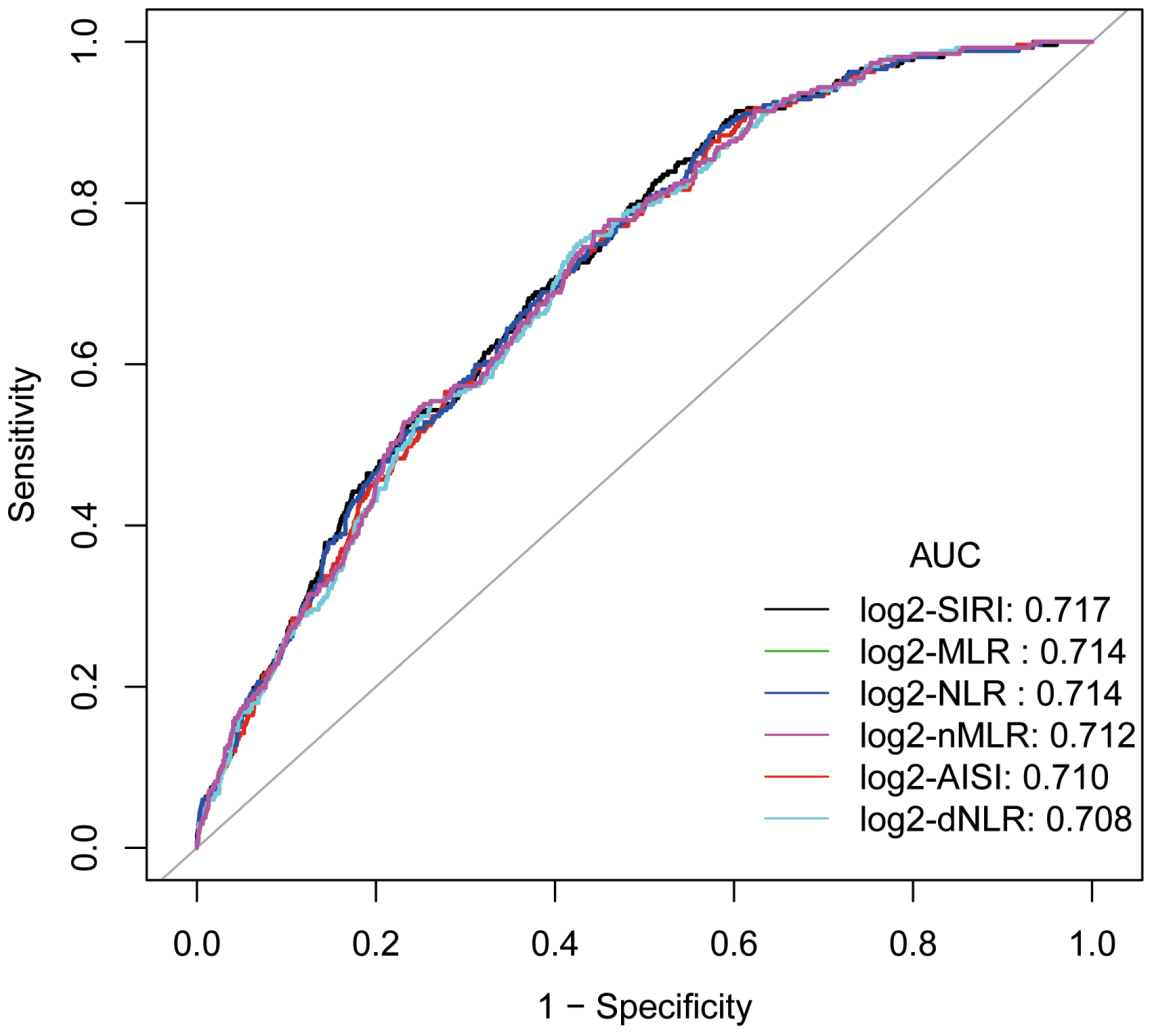
ROC curve and Area under curve (AUC).Adjusted for age, race/ethnicity, income, education, drinking status, body mass index (BMI), diabetes mellitus, hypertension, hyperlipidemia, physical activity, serum uric acid, energy intake.

The ROC curve (Figure 3) of the correlation between a SIRI and other inflammatory markers and the prevalence of gout suggests that SIRI has the largest area under the curve (AUC), implying that SIRI is the most accurate inflammatory marker for predicting the prevalence of gout in hyperuricemic female. The AUC of SIRI was 0.717, with a sensitivity of 0.828 and a specificity of 0.488.

### Subgroup analysis

**Figure 4:**
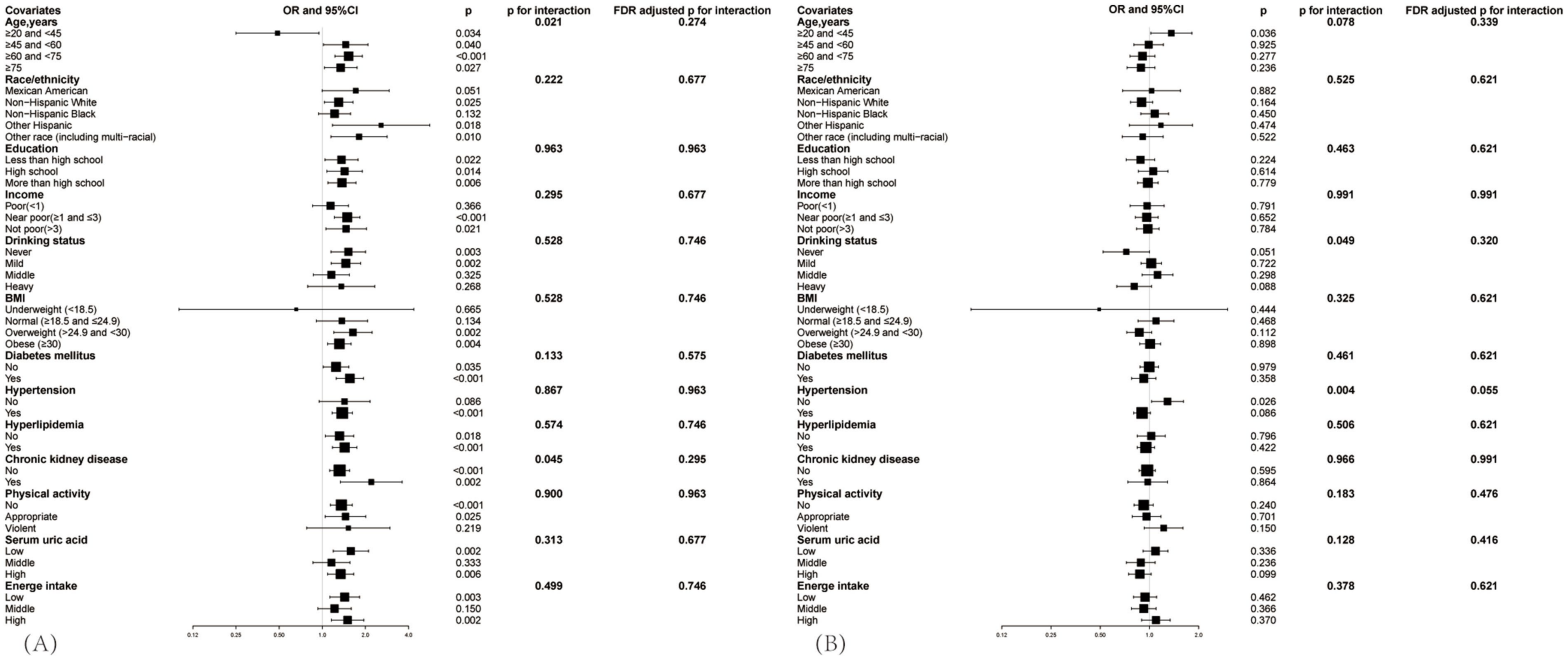
Subgroup analysis between SIRI and the prevalence of gout in hyperuricemic populations, **(A)** is for female, **(B)** is for male. Adjusted for age, race/ethnicity, income, education, drinking status, BMI, diabetes mellitus, hypertension, hyperlipidemia, physical activity, serum uric acid, energy intake. The black square represents the OR value, and the black line indicates the 95% confidence interval (CI) range.

Subgroup analyses were meticulously stratified across a spectrum of variables, including age, race, education level, PIR, BMI, drinking status, diabetes mellitus, hypertension, hyperlipidemia, CKD, physical activity level, serum uric acid and energy intake, to discern the correlation of the SIRI with gout in hyperuricemic female (Figure 4A) and male (Figure 4B). We used the FDR adjustment method to control the false discovery rate in the subgroup analysis. We investigated the heterogeneity within each subgroup using interaction terms, and no significant difference was uncovered (FDR adjusted p for interaction >0.05 for all), revealing that the results were broadly consistent when the individuals were divided into different subgroups.

Though covariates did not significantly impact the correlation, variations were observed between different subgroups. In the hyperuricemic female population (Figure 4A), when aged between 20 and 45 years, log2-SIRI showed a significant negative correlation with gout prevalence [OR = 0.487, 95% CI (0.250, 0.948), *p* = 0.034], whereas in other age groups, the correlation was significantly positive. Compared to patients without CKD [OR = 1.322, 95% CI (1.126, 1.552), *p* < 0.001], those with CKD [OR = 2.200, 95% CI (1.339, 3.613), *p* = 0.002] exhibited a greater increase in the likelihood of gout prevalence with rising SIRI levels. Specifically, the positive correlation between log2-SIRI and gout prevalence was not significant in the non-Hispanic Black, mild drinking, middle drinking, normal weight, without hypertension, middle serum uric acid, and violent physical activity groups (*p* > 0.05). It was negatively correlated in the poor, severe drinking, and underweight groups (*p* > 0.05).

In the hyperuricemic male population (Figure 4B), although the overall correlation between SIRI and gout prevalence was not significant in the hyperuricemic male population, subgroup analysis found a significant positive correlation in the 20–45 age group [OR = 1.362, 95% CI (1.021, 1.818), *p* = 0.036] and among non-hypertensive individuals [OR = 1.291, 95% CI (1.031, 1.615), *p* = 0.026]. In other subgroups, the correlation was not significant (*p* > 0.05).

Subgroup analyses for other inflammatory markers can be found in Supplementary Table S3.

## Discussion

This study aims to analyze the correlation between SIRI and gout prevalence in the hyperuricemic population. We included 6,732 hyperuricemic patients, divided into male and female groups, with 3,764 males, and analyzed the log2-transformed SIRI. Our results show significant differences in SIRI between gout and non-gout populations in both male and female groups (*p* < 0.001).

In the female group, SIRI was significantly positively correlated with gout prevalence [OR = 1.385, 95% CI (1.187, 1.615), *p* < 0.001], and its diagnostic performance was superior to other inflammatory markers. Subgroup analysis revealed that none of the covariates significantly influenced this correlation. However, SIRI had a greater impact on gout prevalence in individuals with CKD, and an opposite relationship was observed between the age groups of 20–45 and over 45 years. In the male group, the overall correlation [OR = 0.992, 95% CI (0.861, 1.143), *p* = 0.912] and other subgroups were not significant, except for the 20–45 age group [OR = 1.362, 95% CI (1.021, 1.818), *p* = 0.036] and the non-hypertensive population [OR = 1.291, 95% CI (1.031, 1.615), *p* = 0.026], where a significant positive correlation was observed.

Previous studies have primarily analyzed the relationship between inflammatory markers derived from complete blood count and acute gout attacks. A clinical study including both male and female participants found that NLR in inter-critical gout patients was significantly higher than in healthy controls, and MLR, NLR, and PLR were significantly higher in patients with acute gout compared to those with inter-critical gout (27). Another clinical study focusing solely on male patients during acute and attack-free periods of gout found that SIRI was associated with acute gout attacks and had better diagnostic performance than SII, MLR, NLR, and PLR, suggesting that SIRI could be used as a novel inflammatory marker for disease activity in gouty arthritis (26). This is consistent with our findings that SIRI showed significant differences between gout and non-gout groups and had superior diagnostic performance compared to other markers. However, our study specifically focused on the correlation between SIRI and gout prevalence in the hyperuricemic population, and due to the gender differences in gout prevalence, we conducted separate analyses for each gender. We propose that the following mechanisms might explain this correlation.

Both gout remission and flares are associated with immune cells and various immune factors (30). MSU crystals interact with resident macrophages to trigger the activation of the NLRP3 inflammasome, which subsequently activates caspase 1, leading to the processing and production of bioactive IL-1β (31). The signaling of IL-1β stimulates the production and release of pro-inflammatory mediators, which attract neutrophils to the joint cavity, where they release large amounts of pro-inflammatory mediators, thereby intensifying the inflammatory response in the joint (2). Neutrophils may also play a role in resolving inflammation during a gout flare by enhancing the production of anti-inflammatory mediators (32)or pro-resolving lipids (33), which help to downregulate joint inflammation. Moreover, in the later stages of a gout flare, dying neutrophils release their chromosomal DNA, forming structures known as neutrophil extracellular traps (NETs), a process termed NETosis. Aggregated NETs (aggNETs) capture and neutralize several pro-inflammatory mediators (34), leading to the resolution of the gout flare.

It can be seen from above that gout attack is mediated principally by macrophages and neutrophils (30). The SIRI formula involves neutrophils and monocytes (macrophages). In systemic inflammation, lymphocytes decrease as the number of neutrophils increases (35). Therefore, SIRI comprehensively reflects the balance between the inflammatory response and the immune status in gout patients. In addition to SIRI, both nMLR and AISI also incorporate neutrophils, lymphocytes, and monocytes. However, the calculation of nMLR utilizes addition rather than multiplication, which may influence its sensitivity to gout.

Additionally, AISI includes platelet counts in its calculation. Although platelets can exacerbate inflammation by adhering to endothelial cells and facilitating leukocyte migration to affected areas (36), their role in the pathophysiology of gout is not prominent. This inclusion may compromise the diagnostic accuracy of AISI. Other indices that do not account for both neutrophils and monocytes might explain why SIRI demonstrates superior diagnostic efficacy.

There are significant differences in the association between the Systemic Inflammation Response Index (SIRI) and gout prevalence among individuals with hyperuricemia across different age groups and genders. The following explores potential reasons for these variations. In women aged 20–45 with hyperuricemia, SIRI is significantly negatively correlated with gout prevalence. This association may be attributed to higher estrogen levels in this age group, as estrogen can reduce serum uric acid levels and lower gout risk through mechanisms such as promoting uric acid excretion (37–39), exerting anti-inflammatory effects (40), and stimulating antioxidant enzymes to regulate oxidative stress (41). However, in women over 45, the protective effect of estrogen diminishes due to its decline, leading to increased levels of pro-inflammatory factors such as IL-1 (42)and a higher risk of gout (43) in postmenopausal women. This may explain the significant positive correlation between SIRI and gout prevalence in hyperuricemic women in this age group.

In men aged 20–45 with hyperuricemia, SIRI is significantly positively correlated with gout prevalence. This may be related to higher androgen levels in younger men, which increase metabolic rates (44). Additionally, higher alcohol consumption among hyperuricemic men, as shown in Table 1 (45–47), may also contribute to this association. These factors could elevate uric acid production and inflammation levels, thereby increasing the risk of gout. In contrast, for men over 45, decreased androgen levels, reduced metabolic rate, increased body weight, and comorbid chronic diseases (48) may influence gout prevalence (49–52) or SIRI levels (20, 53–55), leading to a non-significant correlation between SIRI and gout in this age group.

Moreover, in the female group with CKD, an elevated log2-SIRI significantly increases the likelihood of developing gout. Previous studies have shown that the prevalence of gout increases with declining renal function (52). This is likely because approximately two-thirds of urate is excreted through the kidneys (56), leading to greater fluctuations in serum uric acid levels in CKD patients due to changes in diet, medication, and other factors, thereby triggering gout. In the male group, the impact of hypertension on this correlation might be influenced by antihypertensive medications (51). Calcium channel blockers and losartan are associated with a lower risk of incident gout, while angiotensin-converting enzyme inhibitors, diuretics, *β* blockers, and non-losartan angiotensin II receptor blockers are associated with an increased risk of gout.

The main findings of this study provide valuable insights for clinical practice and future research. Our analysis indicates that the Systemic Inflammation Response Index (SIRI) is associated with gout prevalence in hyperuricemic women, suggesting its potential as a predictive marker. Compared to the lack of specificity in risk factors and the high cost of genetic testing, SIRI is easily obtainable, relatively specific, and highly applicable. Future research should focus on prospective studies, large-scale investigations, or fundamental research to further explore the predictive role of SIRI. If its predictive capacity is confirmed, SIRI could be used as a preliminary screening tool to identify high-risk individuals for gout among those with hyperuricemia. Combined with strategies to reduce risk factors and interventions such as fenofibrate (57), febuxostat (58), and the IL-1β-targeting monoclonal antibody canakinumab (59), this approach could aid in developing preventive measures to reduce the incidence of gout.

Compared to previous studies, our research has several advantages. To our knowledge, this is the first study to analyze the correlation between gout prevalence and the systemic inflammation response index (SIRI) in a population with hyperuricemia. It also comprehensively evaluates the relationship between SIRI and gout by comparing it with other inflammatory markers. Second, we ensured the robustness and reliability of our results by adjusting for confounding factors, conducting subgroup analyses, and performing a straightforward sensitivity analysis by converting SIRI into quartile variables. Additionally, our study addresses the differences in gout prevalence between males and females by performing separate correlation analyses for each gender.

However, there are some limitations to this study. Firstly, considering the high prevalence in men, the potential of SIRI as a predictive marker for gout risk is limited. Then, due to the cross-sectional design of NHANES, we were unable to determine the predictive role and causality of SIRI on the attack of gout in a hyperuricemic population. Future research could adopt longitudinal study designs or utilize more advanced analytical methods (such as structural equation modeling or causal inference techniques) to explore the causal relationships between variables further. In addition, our analyses were based on the U.S. population collected from the NHANES dataset, which may lead to a lack of applicability to non-U.S. populations and require data from other countries or regions for validation and supplementation. Future research should utilize broader datasets from other regions or conduct multinational studies to verify whether our findings are cross-culturally applicable. Finally, the included covariates do not fully summarize the influences that may affect the development of gout in the hyperuricemia population in actual clinical practice, and other influences that were not included may bias our results to some extent, such as medical conditions, occupational status, and genetic factor.

## Conclusion

Our study suggests that the SIRI is significantly positively associated with gout prevalence in hyperuricemic women but not in men. Given the higher prevalence of gout in men, the potential of SIRI as a predictive marker for gout risk in this population may be limited. However, subgroup analyses indicated that the relationship between SIRI and gout prevalence and its statistical significance varied across different age groups. Future studies could explore this association further by investigating the relationship between SIRI and gout prevalence in various age cohorts. Large-scale prospective studies, as well as research utilizing diverse datasets from different regions or countries, could further strengthen the validity and generalizability of these findings.

## Data Availability

Publicly available datasets were analyzed in this study. This data can be found here: http://www.cdc.gov/nchs/nhanes/.
