# Supplementary material for: Systemic inflammation response index association with gout in hyperuricemic adults: NHANES 2007–2018
Source: Front Med (Lausanne). 2025 Jan 7;11:1490655. doi: 10.3389/fmed.2024.1490655 (PMC11752896; doi:10.3389/fmed.2024.1490655)
Supplement: Supplementary file 1 [file Table_1.DOCX]

Supplementary Table 1: Normality Test

|  | Male | | | Female | | |
| --- | --- | --- | --- | --- | --- | --- |
| log2-SIRI | Anderson-Darling normality test | 202.1836 | <0.001 | Anderson-Darling normality test | 135.6936 | <0.001 |
|  | Cramer-von Mises normality test | 35.049 | <0.001 | Cramer-von Mises normality test | 23.1049 | <0.001 |
|  | Lilliefors (Kolmogorov-Smirnov) normality test | 0.1454 | <0.001 | Lilliefors (Kolmogorov-Smirnov) normality test | 0.135 | <0.001 |
|  | Pearson chi-square normality test | 1841.5733 | <0.001 | Pearson chi-square normality test | 1239.3396 | <0.001 |
|  | Shapiro-Francia normality test | 0.6708 | <0.001 | Shapiro-Francia normality test | 0.7527 | <0.001 |
| log2-SII | Anderson-Darling normality test | 405.7136 | <0.001 | Anderson-Darling normality test | 111.352 | <0.001 |
|  | Cramer-von Mises normality test | 72.4959 | <0.001 | Cramer-von Mises normality test | 18.6967 | <0.001 |
|  | Lilliefors (Kolmogorov-Smirnov) normality test | 0.2296 | <0.001 | Lilliefors (Kolmogorov-Smirnov) normality test | 0.1226 | <0.001 |
|  | Pearson chi-square normality test | 3589.5377 | <0.001 | Pearson chi-square normality test | 946.1651 | <0.001 |
|  | Shapiro-Francia normality test | 0.3186 | <0.001 | Shapiro-Francia normality test | 0.7504 | <0.001 |
| log2-AISI | Anderson-Darling normality test | 458.7727 | <0.001 | Anderson-Darling normality test | 163.3796 | <0.001 |
|  | Cramer-von Mises normality test | 84.1808 | <0.001 | Cramer-von Mises normality test | 28.4691 | <0.001 |
|  | Lilliefors (Kolmogorov-Smirnov) normality test | 0.2578 | <0.001 | Lilliefors (Kolmogorov-Smirnov) normality test | 0.1463 | <0.001 |
|  | Pearson chi-square normality test | 4621.7412 | <0.001 | Pearson chi-square normality test | 1521.2877 | <0.001 |
|  | Shapiro-Francia normality test | 0.2989 | <0.001 | Shapiro-Francia normality test | 0.7036 | <0.001 |
| log2-PLR | Anderson-Darling normality test | 95.8589 | <0.001 | Anderson-Darling normality test | 58.8857 | <0.001 |
|  | Cramer-von Mises normality test | 15.9516 | <0.001 | Cramer-von Mises normality test | 9.8086 | <0.001 |
|  | Lilliefors (Kolmogorov-Smirnov) normality test | 0.1047 | <0.001 | Lilliefors (Kolmogorov-Smirnov) normality test | 0.0942 | <0.001 |
|  | Pearson chi-square normality test | 818.3507 | <0.001 | Pearson chi-square normality test | 498.0283 | <0.001 |
|  | Shapiro-Francia normality test | 0.854 | <0.001 | Shapiro-Francia normality test | 0.8684 | <0.001 |
| log2-MLR | Anderson-Darling normality test | 116.9039 | <0.001 | Anderson-Darling normality test | 92.3025 | <0.001 |
|  | Cramer-von Mises normality test | 19.6904 | <0.001 | Cramer-von Mises normality test | 15.7759 | <0.001 |
|  | Lilliefors (Kolmogorov-Smirnov) normality test | 0.1392 | <0.001 | Lilliefors (Kolmogorov-Smirnov) normality test | 0.1257 | <0.001 |
|  | Pearson chi-square normality test | 1765.933 | <0.001 | Pearson chi-square normality test | 1407.8278 | <0.001 |
|  | Shapiro-Francia normality test | 0.8159 | <0.001 | Shapiro-Francia normality test | 0.8407 | <0.001 |
| log2-NLR | Anderson-Darling normality test | 170.6425 | <0.001 | Anderson-Darling normality test | 102.6026 | <0.001 |
|  | Cramer-von Mises normality test | 29.103 | <0.001 | Cramer-von Mises normality test | 17.2049 | <0.001 |
|  | Lilliefors (Kolmogorov-Smirnov) normality test | 0.1368 | <0.001 | Lilliefors (Kolmogorov-Smirnov) normality test | 0.1192 | <0.001 |
|  | Pearson chi-square normality test | 1527.0765 | <0.001 | Pearson chi-square normality test | 845.9198 | <0.001 |
|  | Shapiro-Francia normality test | 0.6998 | <0.001 | Shapiro-Francia normality test | 0.7589 | <0.001 |
| log2-PPN | Anderson-Darling normality test | 575.6296 | <0.001 | Anderson-Darling normality test | 72.4623 | <0.001 |
|  | Cramer-von Mises normality test | 106.5219 | <0.001 | Cramer-von Mises normality test | 12.173 | <0.001 |
|  | Lilliefors (Kolmogorov-Smirnov) normality test | 0.2788 | <0.001 | Lilliefors (Kolmogorov-Smirnov) normality test | 0.1057 | <0.001 |
|  | Pearson chi-square normality test | 5444.4118 | <0.001 | Pearson chi-square normality test | 684.2547 | <0.001 |
|  | Shapiro-Francia normality test | 0.1743 | <0.001 | Shapiro-Francia normality test | 0.8597 | <0.001 |
| log2-dNLR | Anderson-Darling normality test | 94.5199 | <0.001 | Anderson-Darling normality test | 59.4345 | <0.001 |
|  | Cramer-von Mises normality test | 15.7745 | <0.001 | Cramer-von Mises normality test | 9.6563 | <0.001 |
|  | Lilliefors (Kolmogorov-Smirnov) normality test | 0.1011 | <0.001 | Lilliefors (Kolmogorov-Smirnov) normality test | 0.0875 | <0.001 |
|  | Pearson chi-square normality test | 828.7088 | <0.001 | Pearson chi-square normality test | 519.2618 | <0.001 |
|  | Shapiro-Francia normality test | 0.8102 | <0.001 | Shapiro-Francia normality test | 0.843 | <0.001 |
| log2-nMLR | Anderson-Darling normality test | 166.2738 | <0.001 | Anderson-Darling normality test | 100.5341 | <0.001 |
|  | Cramer-von Mises normality test | 28.324 | <0.001 | Cramer-von Mises normality test | 16.9584 | <0.001 |
|  | Lilliefors (Kolmogorov-Smirnov) normality test | 0.1361 | <0.001 | Lilliefors (Kolmogorov-Smirnov) normality test | 0.1194 | <0.001 |
|  | Pearson chi-square normality test | 1469.771 | <0.001 | Pearson chi-square normality test | 865.5307 | <0.001 |
|  | Shapiro-Francia normality test | 0.7137 | <0.001 | Shapiro-Francia normality test | 0.7703 | <0.001 |
