# Supplementary material for: Systemic inflammation response index association with gout in hyperuricemic adults: NHANES 2007–2018
Source: Front Med (Lausanne). 2025 Jan 7;11:1490655. doi: 10.3389/fmed.2024.1490655 (PMC11752896; doi:10.3389/fmed.2024.1490655)
Supplement: Supplementary file 2 [file Table_2.DOCX]

**Supplementary Table 2**

**Covariates**

**Age** was classified as 4 levels(≥20 and <45,≥45 and <60,≥60 and <75,≥75) based on WHO classification.

**Race** was categorized into Mexican American, non-Hispanic Black, non-Hispanic White, other Hispanic, and other race (including multi-racial) based on NHANES classification.

**Education level** was classified as less than high school, high school graduate and more than high school[1].

**PIR** was categorized into three strata (<1, 1–3, and ≥3) and respectively defined as poor, near poor, and not poor[2].

**BMI** was considered as underweight( <18.5 ),normal (18.5–24.9), overweight (25–29.9), and obesity(≥30)[3].

**Drinking status** was defined as never for who answered no to “Had at least 12 alcohol drinks/lifetime?”. If the answer is year, drinking status was defined by the answer to “In the past 12 months, on those days that you drank alcoholic beverages, on the average, how many drinks did you have?”. The definition criteria is different for male and female. For female,≤1 drink per day defined as mild drinking,(1–3 drinks per day defined as middle drinking,≥4 drinks per day defined as heavy drinking. For male, ≤2 drink per day defined as mild drinking,(3–4 drinks per day defined as middle drinking,≥5 drinks per day defined as heavy drinking[4].

**Diabetes mellitus** (DM) was defined as self-reported diabetes, or the use of diabetes medication or insulin or glycohemoglobin (HbA1c) >6.5%, or fasting glucose ≥7.0 mmol/L, or random blood glucose ≥11.1 mmol/L, or 2h oral glucose tolerance test (OGTT) blood glucose (mmol/L) ≥11.1[5].

**Hypertension**(HBP) was defined as self-reported hypertension, or the use of prescribed medicine for HBP , or systolic blood pressure≥140 mmHg and/or diastolic blood pressure ≥90 mmHg based on the average of three measurements of all subjects’ blood pressure[6].

**Hyperlipidemia** was defined as self-reported high cholesterol level or total cholesterol 200 mg/dL, triglycerides 150 mg/dL, HDL 40 mg/dL in males and 50 mg/dL in females, or low-density lipoprotein 130 mg/dL[7].

**Chronic kidney disease:** We used the Chronic Kidney Disease Epidemiology Collaboration formulate to assess estimated glomerular filtration rate (eGFR). Chronic kidney disease (CKD) was defined as eGFR <60 ml/min/1.73 m2 or urine albumin creatinine ratio (UACR)>30 mg/g[8].

**Physical activity level:** We defined appropriate physical activity as answer “yes” to “In a typical week, do you do any moderate-intensity sports, fitness, or recreational activities that cause a small increase in breathing or heart rate such as brisk walking, bicycling, swimming, or volleyball for at least 10 min continuously?”, defined sever physical activity as answer “yes” to“In a typical week, do you do any vigorous-intensity sports, fitness, or recreational activities that cause large increases in breathing or heart rate like running or basketball for at least 10 min continuously?”, defined no physical activity as answer “no” to the above two questions[9].

**Energy intake:** The energy intake data were calculated via taking the average of the energy intake in two 24-h dietary recall interviews.

**Serum uric acid:** Uric acid concentration was detected on a Beckman Synchron LX20 (Beckman Coulter, Inc., Brea, CA) using a colorimetric method[11].

**References**

1. Ikonte CJ, Mun JG, Reider CA, Grant RW, Mitmesser SH. Micronutrient Inadequacy in Short Sleep: Analysis of the NHANES 2005–2016. Nutrients. 2019;11:2335.

2. Liu Y. The relationship between lifestyle and self-reported oral health among American adults. Int Dent J. 2014;64:46–51.

3. Lu N, Dubreuil M, Zhang Y, Neogi T, Rai SK, Ascherio A, et al. Gout and the risk of Alzheimer’s disease: a population-based, BMI-matched cohort study. Ann Rheum Dis. 2016;75:547–51.

4. Hicks CW, Wang D, Matsushita K, Windham BG, Selvin E. Peripheral Neuropathy and All-Cause and Cardiovascular Mortality in U.S. Adults : A Prospective Cohort Study. Ann Intern Med. 2021;174:167–74.

5. Zeng G, You D, Ye L, Wu Y, Shi H, Lin J, et al. n-3 PUFA poor seafood consumption is associated with higher risk of gout, whereas n-3 PUFA rich seafood is not: NHANES 2007–2016. Front Nutr. 2023;10:1075877.

6. Li C, Shang S. Relationship between Sleep and Hypertension: Findings from the NHANES (2007–2014). Int J Environ Res Public Health. 2021;18:7867.

7. National Cholesterol Education Program (NCEP) Expert Panel on Detection, Evaluation, and Treatment of High Blood Cholesterol in Adults (Adult Treatment Panel III). Third Report of the National Cholesterol Education Program (NCEP) Expert Panel on Detection, Evaluation, and Treatment of High Blood Cholesterol in Adults (Adult Treatment Panel III) final report. Circulation. 2002;106:3143–421.

8. Kidney Disease: Improving Global Outcomes (KDIGO) Glomerular Diseases Work Group. KDIGO 2021 Clinical Practice Guideline for the Management of Glomerular Diseases. Kidney Int. 2021;100:S1–276.

9. You Y, Chen Y, Fang W, Li X, Wang R, Liu J, et al. The association between sedentary behavior, exercise, and sleep disturbance: A mediation analysis of inflammatory biomarkers. Front Immunol. 2022;13:1080782.

10. Wang J, Chen S, Zhao J, Liang J, Gao X, Gao Q, et al. Association between nutrient patterns and hyperuricemia: mediation analysis involving obesity indicators in the NHANES. BMC Public Health. 2022;22:1981.
