# Supplementary material for: Systemic inflammation response index association with gout in hyperuricemic adults: NHANES 2007–2018
Source: Front Med (Lausanne). 2025 Jan 7;11:1490655. doi: 10.3389/fmed.2024.1490655 (PMC11752896; doi:10.3389/fmed.2024.1490655)
Supplement: Supplementary file 4 [file Table_4.DOCX]

**Sensitivity analysis after excluding outliers of inflammation markers**

| Female | Model 1 | | Model 2 | | Model 3 | |
| --- | --- | --- | --- | --- | --- | --- |
|  | OR(95%CI) | p | OR(95%CI) | p | OR(95%CI) | p |
| log2-SIRI | 1.483 (1.278, 1.721) | <0.001 | 1.461 (1.250, 1.708) | <0.001 | 1.418 (1.206, 1.667) | <0.001 |
| log2-SII | 1.120 (0.948, 1.323) | 0.181 | 1.214 (1.024, 1.439) | 0.025 | 1.164 (0.978, 1.385) | 0.087 |
| log2-AISI | 1.245 (1.092, 1.419) | 0.001 | 1.289 (1.126, 1.475) | 0.000 | 1.244 (1.082, 1.430) | 0.002 |
| log2-PLR | 0.951 (0.748, 1.210) | 0.683 | 0.956 (0.753, 1.215) | 0.716 | 0.986 (0.773, 1.258) | 0.909 |
| log2-MLR | 1.751 (1.411, 2.172) | <0.001 | 1.460 (1.161, 1.836) | 0.001 | 1.470 (1.163, 1.858) | <0.001 |
| log2-NLR | 1.560 (1.285, 1.896) | <0.001 | 1.545 (1.264, 1.889) | <0.001 | 1.509 (1.227, 1.857) | <0.001 |
| log2-PPN | 0.987 (0.836, 1.165) | 0.875 | 1.188 (0.997, 1.415) | 0.054 | 1.101 (0.920, 1.317) | 0.294 |
| log2-dNLR | 1.449 (1.161, 1.807) | 0.001 | 1.533 (1.218, 1.929) | <0.001 | 1.477 (1.166, 1.871) | 0.001 |
| log2-nMLR | 1.630 (1.329, 2.000) | <0.001 | 1.592 (1.289, 1.968) | <0.001 | 1.551 (1.247, 1.929) | <0.001 |

| Male | Model 1 | | Model 2 | | Model 3 | |
| --- | --- | --- | --- | --- | --- | --- |
|  | OR(95%CI) | p | OR(95%CI) | p | OR(95%CI) | p |
| log2-SIRI | 1.190 (1.082, 1.310) | <0.001 | 1.012 (0.910, 1.124) | 0.831 | 0.959 (0.857, 1.073) | 0.464 |
| log2-SII | 1.049 (0.940, 1.170) | 0.391 | 0.996 (0.888, 1.117) | 0.941 | 0.967 (0.857, 1.092) | 0.591 |
| log2-AISI | 1.036 (0.951, 1.128) | 0.422 | 0.978 (0.892, 1.072) | 0.633 | 0.947 (0.860, 1.043) | 0.271 |
| log2-PLR | 1.066 (0.911, 1.247) | 0.423 | 0.937 (0.798, 1.100) | 0.427 | 1.007 (0.851, 1.192) | 0.936 |
| log2-MLR | 1.465 (1.262, 1.700) | <0.001 | 0.957 (0.812, 1.128) | 0.600 | 0.983 (0.828, 1.168) | 0.847 |
| log2-NLR | 1.364 (1.206, 1.542) | <0.001 | 1.097 (0.960, 1.252) | 0.174 | 1.045 (0.907, 1.203) | 0.543 |
| log2-PPN | 0.860 (0.767, 0.964) | 0.009 | 1.004 (0.888, 1.134) | 0.955 | 0.957 (0.841, 1.088) | 0.499 |
| log2-dNLR | 1.049 (0.940, 1.170) | 0.391 | 0.996 (0.888, 1.117) | 0.941 | 0.967 (0.857, 1.092) | 0.591 |
| log2-nMLR | 1.358 (1.190, 1.549) | <0.001 | 1.052 (0.912, 1.213) | 0.489 | 1.002 (0.861, 1.167) | 0.976 |

Supplementary Table 4:Sensitivity analysis after excluding outliers of inflammation markers.

Model 1: no adjustment.

Model 2: adjusted for age, gender, and race/ethnicity. income, education.

Model 3: adjusted for age, gender, race/ethnicity, income, education, drinking status, body mass index(BMI),diabetes mellitus, hypertension, hyperlipidemia, physical activity, serum uric acid, energy intake.

OR, odds ratio; CI, confidence interval; SIRI, systemic inflammatory response index; SII, systemic immune-inflammatory index; AISI,total systemic inflammatory index; PLR, platelet-to-lymphocyte ratio;MLR,monocyte-to-lymphocyte ratio; NLR, neutrophil-to-lymphocyte ratio;PPN,product of platelet count and neutrophil count; dNLR derived neutrophil-to-lymphocyte ratio; nMLR, neutrophil to monocyte plus lymphocyte ratio.

*:p<0.05
