# Supplementary material for: Systemic inflammation response index association with gout in hyperuricemic adults: NHANES 2007–2018
Source: Front Med (Lausanne). 2025 Jan 7;11:1490655. doi: 10.3389/fmed.2024.1490655 (PMC11752896; doi:10.3389/fmed.2024.1490655)
Supplement: Supplementary file 5 [file Table_5.DOCX]

Sensitivity analysis after excluding outliers of serum uric acid

| Female | Model 1 | | Model 2 | | Model 3 | |
| --- | --- | --- | --- | --- | --- | --- |
|  | OR(95%CI) | p | OR(95%CI) | p | OR(95%CI) | p |
| log2-SII | 1.103 (0.943, 1.290) | 0.220 | 1.181 (1.009, 1.383) | 0.038 | 1.158 (0.977, 1.372) | 0.090 |
| log2-AISI | 1.210 (1.067, 1.373) | 0.003 | 1.249 (1.096, 1.423) | 0.001 | 1.217 (1.056, 1.402) | 0.007 |
| log2-SIRI | 1.448 (1.257, 1.668) | <0.001 | 1.423 (1.226, 1.650) | <0.001 | 1.375 (1.169, 1.616) | <0.001 |
| log2-PLR | 0.908 (0.735, 1.124) | 0.376 | 0.925 (0.752, 1.138) | 0.460 | 0.941 (0.755, 1.173) | 0.591 |
| log2-MLR | 1.688 (1.377, 2.069) | <0.001 | 1.413 (1.138, 1.755) | 0.002 | 1.365 (1.082, 1.722) | 0.009 |
| log2-NLR | 1.408 (1.176, 1.686) | 0.000 | 1.376 (1.146, 1.653) | 0.001 | 1.337 (1.098, 1.629) | 0.004 |
| log2-PPN | 0.987 (0.841, 1.159) | 0.875 | 1.174 (0.992, 1.390) | 0.062 | 1.141 (0.950, 1.369) | 0.159 |
| log2-dNLR | 1.326 (1.078, 1.631) | 0.008 | 1.367 (1.107, 1.689) | 0.004 | 1.316 (1.050, 1.650) | 0.017 |
| log2-nMLR | 1.480 (1.225, 1.788) | 0.000 | 1.421 (1.171, 1.724) | 0.000 | 1.380 (1.120, 1.700) | 0.002 |

| Male | Model 1 | | Model 2 | | Model 3 | |
| --- | --- | --- | --- | --- | --- | --- |
|  | OR(95%CI) | p | OR(95%CI) | p | OR(95%CI) | p |
| log2-SII | 1.046 (0.945, 1.157) | 0.388 | 1.012 (0.913, 1.121) | 0.824 | 0.984 (0.881, 1.099) | 0.778 |
| log2-AISI | 1.030 (0.949, 1.118) | 0.478 | 0.981 (0.899, 1.070) | 0.663 | 0.943 (0.858, 1.037) | 0.225 |
| log2-SIRI | 1.177 (1.074, 1.291) | 0.001 | 1.012 (0.915, 1.120) | 0.809 | 0.946 (0.848, 1.056) | 0.324 |
| log2-PLR | 1.020 (0.882, 1.179) | 0.792 | 0.921 (0.798, 1.063) | 0.261 | 0.984 (0.845, 1.147) | 0.841 |
| log2-MLR | 1.356 (1.181, 1.557) | 0.000 | 0.917 (0.789, 1.065) | 0.255 | 0.903 (0.769, 1.060) | 0.212 |
| log2-NLR | 1.294 (1.152, 1.454) | 0.000 | 1.072 (0.951, 1.208) | 0.256 | 1.011 (0.889, 1.150) | 0.868 |
| log2-PPN | 0.876 (0.787, 0.975) | 0.015 | 1.018 (0.908, 1.141) | 0.765 | 0.961 (0.849, 1.088) | 0.530 |
| log2-dNLR | 1.255 (1.096, 1.437) | 0.001 | 1.100 (0.959, 1.262) | 0.171 | 1.024 (0.885, 1.185) | 0.753 |
| log2-nMLR | 1.329 (1.175, 1.504) | <0.001 | 1.053 (0.924, 1.202) | 0.438 | 0.989 (0.858, 1.141) | 0.880 |

Supplementary Table 5:Sensitivity analysis after excluding outliers of serum uric acid

Model 1: no adjustment.

Model 2: adjusted for age, gender, and race/ethnicity. income, education.

Model 3: adjusted for age, gender, race/ethnicity, income, education, drinking status, body mass index(BMI),diabetes mellitus, hypertension, hyperlipidemia, physical activity, serum uric acid, energy intake.

OR, odds ratio; CI, confidence interval; SIRI, systemic inflammatory response index; SII, systemic immune-inflammatory index; AISI,total systemic inflammatory index; PLR, platelet-to-lymphocyte ratio;MLR,monocyte-to-lymphocyte ratio; NLR, neutrophil-to-lymphocyte ratio;PPN,product of platelet count and neutrophil count; dNLR derived neutrophil-to-lymphocyte ratio; nMLR, neutrophil to monocyte plus lymphocyte ratio.

*:p<0.05
